# Clinical Efficacy of Biosimilar Switch of Adalimumab for Management of Uveitis

**DOI:** 10.1080/09273948.2023.2172591

**Published:** 2023-02-21

**Authors:** G. M. Murray, N. Griffith, P. Sinnappurajar, D. A. Al Julandani, S. L. N. Clarke, D. P. Hawley, J. Choi, C. M. Guly, Athimalaipet V. Ramanan

**Affiliations:** a Department of Rheumatology, Bristol Royal Hospital for Children, Bristol, UK; b Department of Ophthalmology Sheffield Teaching Hospital NHS Foundation Trust, Sheffield, UK; c MRC Integrative Epidemiology Unit and School of Population Health Sciences, University of Bristol, Bristol, UK; d Department of Rheumatology Sheffield Children’s NHS Foundation Trust, Sheffield, UK; e Department of Ophthalmology University Hospitals Bristol and Weston NHS Foundation Trust, Bristol, UK; f Bristol Medical School: Translational Health Sciences, University of Bristol, Bristol, UK

**Keywords:** Adalimumab, biosimilar, clinical efficacy, TNF-alpha, uveitis

## Abstract

**Background:**

Adalimumab has demonstrated efficacy in non-infectious uveitis. With the introduction of biosimilar agents such as Amgevita, we aimed to quantify efficacy and tolerability compared to Humira in a multi-centre UK cohort

**Methods:**

Patients identified from tertiary uveitis clinics in 3 centres, after institution-mandated switching was implemented.

**Results:**

Data collected for 102 patients, aged 2–75 years, with 185 active eyes. Following switch, rates of uveitis flare were not significantly different (13 events before, 21 after, *p* = .132). Rates of elevated intraocular pressure were decreased (32 before, 25 afterwards, *p* = .006) and dosing of oral and intra-ocular steroids was stable. Twenty-four patients (24%) requested to return to Humira, commonly due to pain from injection or technical difficulty with the device.

**Conclusion:**

Amgevita is safe and effective for inflammatory uveitis with non-inferiority to Humira. Significant numbers of patients requested to switch back due to side effects including injection site reactions.

Adalimumab has been shown to be effective in the management of inflammatory uveitis and so has become widely used.^1–3^ However, it is expensive, costing the National Health Service in the United Kingdom over three hundred million pounds in 2016–2017 to treat 57,000 patients.^4^ Following the end of patent, and the development of multiple biosimilar medications, the NHS implemented non-medical switching, i.e. switching to a cheaper biosimilar for economic reasons, for patients receiving originator adalimumab (known as Humira). This is an emerging change in Europe, Australia, and Korea.^5,6^ Biosimilar medications, unlike generic versions of conventional medications, are not identical to the original drug, since both are large and complex molecules.^7^

Theoretical concerns when switching to biosimilars include loss of efficacy, changes in immunogenicity and differences in safety compared with the originator medication.^8^ However, there are limited trials to compare these medications in children and young people. In adult data, there have been reports of ‘failed switches’ where patients request to switch back to the originator drug, frequently due to non-specific side effects. These are thought to be related to individual’s negative expectations of the switch.^9–11^ However, failed switching can have important clinical implications including exhaustion of therapeutic options, risk of exposure to other medications, worse overall patient outcomes, as well as increased health-care costs.

## Aims

This study aimed to evaluate the clinical efficacy of the biosimilar switch of adalimumab in paediatric and adult uveitis patients.

### Methods

Patients with a diagnosis of uveitis, who had switched from originator adalimumab (Humira) to a biosimilar (Amgevita) were included. Patients were retrospectively identified from three tertiary centres in the United Kingdom: paediatric uveitis clinics in Bristol Eye Hospital and Sheffield Children’s Hospital, and the adult uveitis clinic in Sheffield Teaching Hospital. Recruitment occurred over a 6-month period in late 2019-mid 2020. The study was performed shortly after a mandatory change within each institution from Humira to Amgevita.

To evaluate the clinical efficacy of this biosimilar switch, patient data were compiled and analysed. This included demographic and clinical data such as age at diagnosis, disease laterality, presence of underlying systemic disease, ocular comorbidities, and structural complications. These included elevated intra-ocular pressure, defined as >21 mmHg, and hypotony defined as <5 mmHg, visual impairment as recorded in logMAR (with significant impairment as either worse than 0.3, or 1.0 or worse), macular oedema defined as intraretinal cysts on ocular coherence tomography (OCT), presence of posterior synechiae were documented.^12^ Anterior chamber cell score using the standardised uveitis nomenclature (SUN).^12^ Pharmacological and surgical treatments were documented. The patient data were also reviewed for routine visits within the period of six months before and six months after the switch. The appointment closest to the time interval of 2, 4, and 6 months was selected for analysis. Variables including visual acuity, intra ocular pressure, and use of steroid drops were recorded at each visit.

Statistical analyses were performed using Microsoft Excel (version 2003). Comparisons were undertaken using Chi2 for categorical variables and two-tailed paired t-test for continuous variables.

## Results

One hundred and two patients who had switched from Humira to Amgevita (ABP501) were included. Patients who had stopped Humira due to disease remission and restarted on Amgevita due to a flare of disease, within 6 months, were included. The key demographic results of the cohort are outlined in Table 1. Use of other Disease Modifying Anti-Rheumatic Drugs (DMARDs) and pre-existing complications for the cohort are presented as supplementary data (Tables S3 and S4).

**Table 1.** Demographic data.

	Whole Cohort (n = 102)	Paediatric Cohort (n = 67)	Adult Cohort (n = 35)
Gender (F, %)	61 (60)	43 (64)	18 (51)
Age (years)			
Median	7.5	5.4	38.67
Range	2-75	2-13.6	15.92-75
Uveitis Type (n, %)			
Anterior	70 (68.6)	61	9
Intermediate	7 (6.9)	5	2
Posterior	10 (9.8)	-	10
Pan uveitis	15 (14.7)	1	14
Active Eyes: (n, %)	185	121	64
Bilateral	83 (81)	54 (81)	29 (83)
Unilateral	19 (19)	13 (19)	6 (17)
Underlying diagnosis (n, %)			
Idiopathic	23 (22.5)	16 (24)	7 (20)
JIA	49 (48)	49 (73)	0 (0)
Birdshot	7 (6.9)	-	7 (20)
Sarcoid	3 (2.9)	-	3 (9)
Behcet	3 (2.9)	1 (1.5)	2 (6)
Other*	17 (16.7)	1 (1.5)	16 (46)
Duration of follow up (years)			
Median	5.92	4.75	8.25
Range	1.33-21.25	1.33-12.17	1.92-21.25

^a^Includes rheumatoid arthritis related, CNS vasculitis related, JIA = juvenile idiopathic arthritis.

All paediatric patients had been treated with methotrexate, though 17 patients (25%) had stopped due to side effects, most often gastrointestinal related such as nausea. Fifteen of these 17 (88%) were switched to an alternate DMARD, mycophenolate mofetil. Of the paediatric patients, 21/67 (31%) had had periods of time off Humira but had flares of eye disease necessitating re-commencement.

## Post-switch findings: Clinical efficacy and patient reported issues

Following the switch from to Amgevita, there was 1 event of worsening visual acuity (logMar >0.3 worsening to >1.0), giving an incidence rate of 0.0197/Eye Year. There were 2 episodes of a significant improvement in visual acuity (from worse than logMar 1.0 to 0.3 logMar), and 7 episodes of patients with visual acuity (logMar >0.3) which improved to <0.3 logMar.

Post-switch there was no significant difference in the number of patients who flared, or the dose of oral or topical glucocorticoid used (Table 2). There was a significant difference in the number of patients with raised IOP between the two groups; with fewer patients in the post-switch group having raised IOP compared to the pre-switch group (Table 2).

**Table 2.** Comparison of pre- and post-switch clinical findings.

	Pre-switch	Post-switch	Chi^2^, *P* value
Number of eyes assessed	185 (100%)	185 (100%)	
Anterior chamber cell count			
Two step increase in inflammation	13 0.33/EY	21 0.41/EY	0.132
Intraocular pressure			7.51
>21mmHg	32	25	0.006
Number of glucocorticoid drops per eye per day (median of 3 visits, per eye)			0.144
</=3	79	76	0.144
>3	4	5	0.07
Data missing or patient switched back to Humira	19	21	
Number of patients on oral prednisolone	24	25	0.031
mean dose (mg)	5	5	0.85
InterQuartile Range	0-7.75	0-6.67	0.12

In comparing flares of disease, there was no significant difference identified. Many of the flares in both periods were mild. In the pre-switch period 1 patient had bilateral flare, compared to 3 patients in the post-switch period. One patient self-ceased their adalimumab and had a flare noted at the subsequent clinic visit.

Following the switch to Amgevita 20 patients reported side effects. Increased pain from the injection was the commonest reported side effect (13/20, 65% of patients, of whom 12/13 (92.3%) were paediatric age group). Other side effects included localised skin reactions, patient reported decreased vision and technical issues with the injector.

Of the 102 patients, 24, (24%) switched back to Humira (15 paediatric, 9 adults). These were at the patient’s request (for pain or technical difficulties with injector) in all but one patient, who had an allergic reaction to the biosimilar. Four patients were deemed to have treatment failure on adalimumab, and switched to an alternate agent (tocilizumab, baricitinib).

## Discussion

We report, to our knowledge, the largest cohort of the effects of biosimilar switch to amgevita in uveitis.

From our data it appears that Amgevita is similarly efficacious to Humira in managing disease activity and minimising complications associated with uveitis. This real-world example of a heterogenous cohort of uveitis patients provides valuable information on the practical use of adalimumab and this biosimilar, and is in keeping with other published case series examining biosimilar use in non-infectious uveitis.

Fabiani et al. examined ocular flare rates in 37 patients with non-infectious uveitis pre- and post-switch from a variety of anti-TNFa bio-originators to their respective biosimilars.^13^ They found no statistically significant difference in the 12 months following switching. They also found that the probability of experiencing ocular flare after the switch appeared independent of disease control prior to the switch. No severe adverse events were observed following the switch.

A study of 89 paediatric patients with a variety of autoimmune conditions, of whom 13 had uveitis, showed no statistically significant differences in efficacy between the bio-originator and biosimilar adalimumab groups after 3, 6, and 12 months.^14^ The same biosimilar (Amgevita) was used. In our study, which covered a shorter period pre and post-switch, 4 patients flared in both periods, suggesting they had fundamentally difficult to control disease and that adalimumab (in either bio-originator or biosimilar form) was insufficient to manage their disease.

In a retrospective study of 26 adults with uveitis, the adalimumab biosimilar, Imraldi/SB5, was effective in significantly reducing uveitis relapses and the occurrence of retinal vasculitis.^15^ Moreover, SB5 biosimilar improved visual acuity, allowed a significant glucocorticoid-sparing effect and showed an excellent drug retention rate. Although the rates of oral glucocorticoids remained stable in our study, there were 9 events of improved visual acuity. In a study aiming to demonstrate effectiveness of another biosimilar adalimumab, CinnoRA, in treating Behçet’s uveitis in 48 patients, a significant improvement in visual acuity and anterior chamber cell grade after adalimumab biosimilar use was found and the vitreous haze grade decreased significantly (*p*-value<.001) with significant corticosteroid-sparing effect (*p*-value = .03).^16^

A case series of 46 patients with JIA, who were commenced on biosimilar Amgevita, found 86.9% of patients achieved complete remission.^17^ The study demonstrated a statistically significant decrease in Juvenile Arthritis Disease Activity Score (JADAS27) scores across 12 months, as well as uveitis remission in the 9 patients who had uveitis.

In a cohort study by Loft et al., drug retention rates were assessed following a mandatory non-medical switch from adalimumab originator to biosimilar in patients with psoriasis (348 in the biosimilar cohort and 378 patients in the bio-originator cohort).^18^The 1-year drug retention rates were similar between the 2 groups (92.0% versus 92.1%) and non-significant differences in hazard ratios for all cause discontinuation, insufficient effect, and adverse events were observed

## Switching back to bio-originator

For almost all patients switching back to Humira in our study, it was at the individual’s request due to discomfort from the new injection device. The documented reason for switch back to bio-originator was never related to a flare in uveitis disease activity, and in only 1 case it was initiated by the treating clinician. The patient developed acute arm pain 24 hours following Amgevita administration, suggesting an allergic reaction, and was switched back to originator Humira with no further issues. For patients with substantial flares, the treating clinicians elected to change to an alternate biologic agent to control uveitis.

Previous evaluation of patient opinion around switching has documented patient and parent expressed concerns about drug administration, particularly whether the formulation would sting (related to presence of citrate, which Humira initially contained), which is an important consideration in paediatric populations.^11^ Of particular note, the biosimilar preparation (Amgevita) used in our patients was citrate free but contained an increased volume of medication (0.9 ml vs 0.4 ml in bio-originator Humira) and also had a slightly larger gauge needle which may account for some reports of increased pain with the biosimilar injection.

For patients, switching from a product with a known individual response, to a new unknown option, exposes them to unpredictable risks.^13^ Qualitative studies amongst patients undergoing non-medical switching have identified a primary concern being the possible decreased efficacy of a cheaper product.^19^ Our findings of non-inferiority, when added to a growing body of evidence, could help assuage these concerns. However, the high rate of switching back in our study, at 24%, is a concern and engagement with manufacturers may be beneficial.

## Cost

Whilst clinical effectiveness of Humira in uveitis was demonstrated by the VISUAL 1, 2 and Sycamore trials, cost analysis in paediatric uveitis indicated a price reduction of 84% would be required for it to be deemed cost-effective.^1,20–22^ The introduction of biosimilars, with the resultant competition for market share with the bio-originator product, has seen a decrease in the average cost per milligram of a number of monoclonal antibodies, including adalimumab.^23^ Demonstrating clinical equivalence, as we have described in this study, can increase clinician and consumer uptake. This ultimately creates a competitive market, allowing biosimilars to represent a strong driver towards cost-effectiveness of adalimumab in uveitis. This may also affect access to effective biological medications in low- and middle-income countries, where the cost of bio-originator medications has been prohibitively expensive previously.

## Limitations

Challenges which arose during the course of this study included the COVID-19 pandemic, which limited face-to-face appointments for many months. As a result, it was decided to extend the follow up interval post-switch to biosimilar adalimumab, to allow for increased data collection following the worst of the pandemic restrictions and return to face-to-face clinical assessments. Our data collection is also limited by the fact that patient care for the paediatric cohort in Bristol is spread across South-West England and South Wales. Where available, letters from local ophthalmologists were reviewed to allow a more complete dataset.

Other limitations of this study include the retrospective nature, which inherently risks incomplete data collection. We have endeavoured to maximise the available data, however, by completing this study across multiple centres.

## Conclusion

Amgevita, an adalimumab biosimilar is a safe and effective treatment for non-infectious uveitis with similar efficacy to originator adalimumab. These results, while encouraging, are specific to this particular biosimilar, and are not generalisable to other biosimilars currently available on the market. While a significant proportion of patients self-requested to switch back to originator adalimumab, this was most commonly due to injection-related discomfort in the paediatric population. Further research is required into optimising this important clinical consideration.

## Supplementary Material

Supplemental Material
