# Supplementary material for: Clinical Efficacy of Biosimilar Switch of Adalimumab for Management of Uveitis
Source: Ocul Immunol Inflamm. 2023 Feb 21;32(4):442–6. doi: 10.1080/09273948.2023.2172591 (PMC11057845; doi:10.1080/09273948.2023.2172591)
Supplement: Supplemental Material [file IOII_A_2172591_SM0370.zip › IOII_A_2172591_supplemental.docx]

Supplementary Data

Table 3: Other treatments*

|  | **Whole Cohort**  **(n=102)** | **Paediatric Cohort (n=67)** | **Adult Cohort**  **(n=35)** |
| --- | --- | --- | --- |
| Methotrexate   - Ceased due to side effects | 81  17 | 67  17 | 14  0 |
| Mycophenolate mofetil | 51 | 22 | 29 |
| Tacrolimus | 11 | 0 | 11 |
| Azathioprine | 4 | 0 | 4 |
| Infliximab | 5 | 4 | 1 |
| Etanercept | 1 | 1 | 0 |

* Patients could have been treated with more than 1 of these agents

Table 4: Complications (pre-switch)

|  | Whole Cohort  (n=102, 185 eyes) | Paediatric  (n= 67, 121 eyes) | Adult  (n=35, 64 eyes) |
| --- | --- | --- | --- |
| Visual Impairment (number of eyes)   - >0.3 logMAR - >1.0 logMAR | 28  10 | 6  1 | 22  9 (including 1 enucleation) |
| Ocular complication (number of eyes)   - Posterior Synechiae - Cataract - Glaucoma - Macular oedema - Optic disc swelling - Hypotony (<5mmHg) - Band keratopathy - Ocular hypertension(>21mmHg) - Amblyopia - Posterior segment inflammation | 48  64  13  28  8  4  22  24  9  26 | 34  31  6  16  6  1  22  14  7  0 | 14  33  7  12  2  3  0  10  2  26 |
| Ocular procedures   - Cataract - Lens implant - Surgical Posterior Capsulotomy - Vitrectomy - Retinal Detachment Surgery - Removal of band keratopathy - Glaucoma surgery - Steroid Implant - Iridectomy - Laser Capsulotomy - Anti VEGF - Intravitreal steroids (triamcinolone) - Drainage of suprachoroidal haemorrhage | 44  43  6  22  4  5  12  13  4  4  2  11  1 | 12  11  6  10  1  5  5  2  2  1  0  4  0 | 32  32  0  12  3  0  7  11  2  3  2  7  1 |
